# The Proximal Alternating Minimization Algorithm for Two-Block Separable Convex Optimization Problems with Linear Constraints

**DOI:** 10.1007/s10957-018-01454-y

**Published:** 2018-12-24

**Authors:** Sandy Bitterlich, Radu Ioan Boţ, Ernö Robert Csetnek, Gert Wanka

**Affiliations:** 1 Faculty of Mathematics, Chemnitz University of Technology, 09126 Chemnitz, Germany; 2 University of Vienna, Oskar-Morgenstern-Platz 1, 1090 Vienna, Austria; 3 Institute for Numerical and Applied Mathematics, University of Göttingen, 37083 Göttingen, Germany

**Keywords:** Proximal AMA, Lagrangian, Saddle points, Subdifferential, Convex optimization, Fenchel duality, 47H05, 65K05, 90C25

## Abstract

The Alternating Minimization Algorithm has been proposed by Paul Tseng to solve convex programming problems with two-block separable linear constraints and objectives, whereby (at least) one of the components of the latter is assumed to be strongly convex. The fact that one of the subproblems to be solved within the iteration process of this method does not usually correspond to the calculation of a proximal operator through a closed formula affects the implementability of the algorithm. In this paper, we allow in each block of the objective a further smooth convex function and propose a proximal version of the algorithm, which is achieved by equipping the algorithm with proximal terms induced by variable metrics. For suitable choices of the latter, the solving of the two subproblems in the iterative scheme can be reduced to the computation of proximal operators. We investigate the convergence of the proposed algorithm in a real Hilbert space setting and illustrate its numerical performances on two applications in image processing and machine learning.

## Introduction

Tseng introduced in [1] the so-called Alternating Minimization Algorithm (AMA) to solve optimization problems with two-block separable linear constraints and two nonsmooth convex objective functions, one of these assumed to be strongly convex. The numerical scheme consists in each iteration of two minimization subproblems, each involving one of the two objective functions, and of an update of the dual sequence which approaches asymptotically a Lagrange multiplier of the dual problem.

The strong convexity of one of the objective functions allows to reduce the corresponding minimization subproblem to the calculation of the proximal operator of a proper, convex and lower semicontinuous function. This is for the second minimization problem in general not the case; thus, with the exception of some very particular cases, one has to use a subroutine in order to compute the corresponding iterate. This may have a negative influence on the convergence behaviour of the algorithm and affect its computational tractability. One possibility to avoid this is to properly modify this subproblem with the aim of transforming it into a proximal step, and, of course, without losing the convergence properties of the algorithm. The papers [2] and [3] provide convincing evidences for the efficiency and versatility of proximal point algorithms for solving nonsmooth convex optimization problems; we also refer to [4] for a block coordinate variable metric forward–backward method.

In this paper, we address in a real Hilbert space setting a more involved two-block separable optimization problem, which is obtained by adding in each block of the objective a further smooth convex function. To solve this problem, we propose a so-called Proximal Alternating Minimization Algorithm (Proximal AMA), which is obtained by inducing in each of the minimization subproblems additional proximal terms defined by means of positively semidefinite operators. The two smooth convex functions in the objective are evaluated via gradient steps. For appropriate choices of these operators, we show that the minimization subproblems turn into proximal steps and the algorithm becomes an iterative scheme formulated in the spirit of the full splitting paradigm. We show that the generated sequence converges weakly to a saddle point of the Lagrangian associated with the optimization problem under investigation. The numerical performances of Proximal AMA are illustrated in particular in comparison with AMA for two applications in image processing and machine learning.

A similarity of AMA to the classical Alternating Direction Method of Multipliers (ADMM) algorithm, introduced by Gabay and Mercier [5], is obvious. In [6–8] (see also [9, 10]), proximal versions of the ADMM algorithm have been proposed and proved to provide a unifying framework for primal-dual algorithms for convex optimization. Parts of the convergence analysis for the Proximal AMA are carried out in a similar spirit to the convergence proofs in these papers.

## Preliminaries

The convex optimization problems addressed in [1] are of the form

1 $$\begin{aligned}&\inf _{x \in R^n,z \in \mathbb {R}^m} f(x)+g(z) \quad \text {s.t.} \quad Ax +Bz=b, \end{aligned}$$

where $f:\mathbb {R}^n \rightarrow \overline{\mathbb {R}}:=\mathbb {R}\cup \{\pm \infty \}$ is a proper, $\gamma$-strongly convex with $\gamma >0$ (this means that $f-\frac{\gamma }{2}\Vert \cdot \Vert ^2$ is convex) and lower semicontinuous function, $g :\mathbb {R}^m \rightarrow \overline{\mathbb {R}}$ is a proper, convex and lower semicontinuous function, $A \in \mathbb {R}^{r \times n}, B \in \mathbb {R}^{r \times m}$ and $b \in \mathbb {R}^r$.

For $c > 0$, the augmented Lagrangian associated with problem (1), $L_c:\mathbb {R}^n \times \mathbb {R}^m \times \mathbb {R}^r \rightarrow \overline{\mathbb {R}}$ reads

$$\begin{aligned} \quad L_c(x,z,p)=f(x)+g(z)+\langle p, b-Ax-Bz\rangle + \frac{c}{2}\Vert Ax+Bz-b\Vert ^2. \end{aligned}$$

The Lagrangian associated with problem (1) is

$$\begin{aligned} L :\mathbb {R}^n \times \mathbb {R}^m \times \mathbb {R}^r \rightarrow \overline{\mathbb {R}}, \quad L (x,z,p) = f(x)+g(z)+\langle p, b-Ax-Bz\rangle . \end{aligned}$$

Tseng proposed the following so-called Alternating Minimization Algorithm (AMA) for solving (1):

### Algorithm 2.1

*(AMA)* Choose $p^0 \in \mathbb {R}^r$ and a sequence of strictly positive stepsizes $(c_k)_{k\ge 0}$. For all $k \ge 0$, set:

2 $$\begin{aligned} x^k&= {{\,\mathrm{argmin}\,}}_{x \in \mathbb {R}^n}\left\{ f(x)-\langle p^k,Ax\rangle \right\} \end{aligned}$$

3 $$\begin{aligned} z^k&\in {{\,\mathrm{argmin}\,}}_{z \in \mathbb {R}^m}\left\{ g(z)-\langle p^k,Bz\rangle +\frac{c_k}{2} \Vert Ax^k+Bz-b\Vert ^2\right\} \end{aligned}$$

4 $$\begin{aligned} p^{k+1}&=p^k+c_k(b-Ax^k-Bz^k). \end{aligned}$$

The main convergence properties of this numerical algorithm are summarized in the theorem below (see [1]).

### Theorem 2.1

Let $A \ne 0$ and $(x,z) \in {{\,\mathrm{ri}\,}}({{\,\mathrm{dom}\,}}f) \times {{\,\mathrm{ri}\,}}({{\,\mathrm{dom}\,}}g)$ be such that the equality $Ax+Bz=b$ holds. Assume that the sequence of stepsizes $(c_k)_{k \ge 0}$ satisfies

$$\begin{aligned} \epsilon \le c_k \le \frac{2 \gamma }{\Vert A\Vert ^2}-\epsilon \quad \forall k \ge 0, \end{aligned}$$

where $0<\epsilon <\frac{\gamma }{\Vert A\Vert ^2}$. Let $(x^k, z^k, p^k)_{k \ge 0}$ be the sequence generated by Algorithm 2.1. Then there exist $x^* \in \mathbb {R}^n$ and an optimal Lagrange multiplier $p^* \in \mathbb {R}^r$ associated with the constraint $Ax+Bz=b$ such that

$$\begin{aligned} x^k \rightarrow x^*, \quad Bz^k \rightarrow b - Ax^*, \quad p^k \rightarrow p^* (k \rightarrow +\infty ). \end{aligned}$$

If the function $z \mapsto g(z)+\Vert Bz\Vert ^2$ has bounded level sets, then $(z^k)_{k \ge 0}$ is bounded and any of its cluster points $z^*$ provides with $(x^*,z^*)$ an optimal solution of (1).

It is the aim of this paper to propose a proximal variant of this algorithm, called Proximal AMA, which overcomes its drawbacks, and to investigate its convergence properties.

In the remainder of this section, we will introduce some notations, definitions and basic properties that will be used in the sequel (see [11]). Let $\mathcal {H}$ and $\mathcal {G}$ be real Hilbert spaces with corresponding inner products $\langle \cdot , \cdot \rangle$ and associated norms $\Vert \cdot \Vert =\sqrt{\langle \cdot , \cdot \rangle }$. In both spaces, we denote by $\rightharpoonup$ the weak convergence and by $\rightarrow$ the strong convergence.

We say that a function $f:\mathcal {H} \rightarrow \overline{\mathbb {R}}$ is proper, if its domain satisfies the assumption ${{\,\mathrm{dom}\,}}f:=\{x\in \mathcal {H}: f(x)<+\infty \}\ne \emptyset$ and $f(x) > -\infty$ for all $x \in \mathcal {H}$. Let be $\Gamma (\mathcal {H})=\{f:\mathcal {H} \rightarrow \overline{\mathbb {R}}: f \text { is proper, convex and lower semicontinuous} \}$.

The (Fenchel) conjugate function $f^*:\mathcal {H} \rightarrow \overline{\mathbb {R}}$ of a function $f\in \Gamma (\mathcal {H})$ is defined as

$$\begin{aligned} f^*(p)=\text {sup}_{x \in \mathcal {H}}\{\langle p, x \rangle -f(x) \} \quad \forall p \in \mathcal {H}\end{aligned}$$

and is a proper, convex and lower semicontinuous function. It also holds $$f^{**}=f$$, where $$f^{**}$$ is the conjugate function of $$f^*$$. The convex subdifferential of *f* is defined as $$\partial f(x)=\{u\in \mathcal {H}: f(y)\ge f(x)+\langle u,y-x\rangle \forall y\in \mathcal {H}\}$$, if $$f(x) \in \mathbb {R}$$, and as $$\partial f(x) = \emptyset $$, otherwise.

The infimal convolution of two proper functions $f,g:\mathcal{H}\rightarrow \overline{\mathbb {R}}$ is the function $f\Box g:\mathcal{H}\rightarrow \overline{\mathbb {R}}$, defined by $(f\Box g)(x)=\inf _{y\in \mathcal{H}}\{f(y)+g(x-y)\}$.

The proximal point operator of parameter $\gamma$ of *f* at *x*, where $\gamma >0$, is defined as

$$\begin{aligned} \text {Prox}_{\gamma f} : \mathcal{H} \rightarrow \mathcal{H}, \quad \text {Prox}_{\gamma f}(x)={{\,\mathrm{argmin}\,}}_{y \in \mathcal {H}}\left\{ \gamma f(y)+\frac{1}{2}\Vert y-x\Vert ^2\right\} . \end{aligned}$$

According to Moreau’s decomposition formula, we have

$$\begin{aligned} {{\,\mathrm{Prox}\,}}_{\gamma f}(x)+\gamma {{\,\mathrm{Prox}\,}}_{(1/\gamma )f^*}(\gamma ^{-1}x)=x, \ \ \forall x \in \mathcal {H}. \end{aligned}$$

Let $C \subseteq \mathcal {H}$ be a convex and closed set. The strong quasi-relative interior of *C* is

$$\begin{aligned} \text {sqri}(C)=\left\{ x \in C: \cup _{\lambda >0}\lambda (C-x) \text { is a closed linear subspace of } \mathcal {H}\right\} . \end{aligned}$$

We always have ${{\,\mathrm{int}\,}}(C)\subseteq \text {sqri}(C)$, and if $\mathcal {H}$ is finite dimensional, then $\text {sqri}(C)=\text {ri}(C),$ where $\text {ri}(C)$ denotes the interior of *C* relative to its affine hull.

We denote by $S_+(\mathcal {H})$ the set of operators from $\mathcal {H}$ to $\mathcal {H}$ which are linear, continuous, self-adjoint and positive semidefinite. For $M \in S_+(\mathcal {H})$, we define the seminorm $\Vert \cdot \Vert _{M} : \mathcal {H}\rightarrow [0,+\infty )$, $\Vert x\Vert _M= \sqrt{\langle x, Mx \rangle }$. We consider the Loewner partial ordering on $S_+(\mathcal {H})$, defined for $M_1, M_2 \in \mathcal {S}_+(\mathcal {H})$ by

$$\begin{aligned} M_1 \succcurlyeq M_2 \Leftrightarrow \Vert x\Vert _{M_1} \ge \Vert x\Vert _{M_2} ~ \forall x \in \mathcal {H}. \end{aligned}$$

Furthermore, we define for $\alpha >0$ the set $\mathcal {P}_\alpha (\mathcal {H}):=\{M\in \mathcal {S}_+(\mathcal {H}): M \succcurlyeq \alpha \text {Id} \}$, where $\text {Id} : \mathcal {H}\rightarrow \mathcal {H}, \text {Id}(x) = x$ for all $x \in \mathcal {H}$, denotes the identity operator on $\mathcal {H}$.

Let $A:\mathcal {H}\rightarrow \mathcal {G}$ be a linear continuous operator. The operator $A^*:\mathcal {G}\rightarrow \mathcal {H}$, fulfilling $\langle A^*y,x \rangle = \langle y, Ax \rangle$ for all $x \in \mathcal {H}$ and $y \in \mathcal {G}$, denotes the adjoint operator of *A*, while $\Vert A\Vert :=\sup \{\Vert Ax\Vert : \Vert x\Vert \le 1 \}$ denotes the norm of *A*.

## The Proximal Alternating Minimization Algorithm

The two-block separable optimization problem we are going to investigate in this paper has the following formulation.

### Problem 3.1

Let $$\mathcal {H}$$, $$\mathcal {G}$$ and $$\mathcal {K}$$ be real Hilbert spaces, $$f \in \Gamma (H)$$$$\gamma $$-strongly convex with $$\gamma >0$$, $$g \in \Gamma (G)$$, $$h_1:\mathcal {H}\rightarrow \mathbb {R}$$ a convex and Fréchet differentiable function with $$L_1$$-Lipschitz continuous gradient with $$L_1 \ge 0$$, $$h_2:\mathcal {G}\rightarrow \mathbb {R}$$ a convex and Fréchet differentiable functions with $$L_2$$-Lipschitz continuous gradient with $$L_2 \ge 0$$, $$A:\mathcal {H}\rightarrow \mathcal {K}$$ and $$B:\mathcal {G}\rightarrow \mathcal {K}$$ linear continuous operators such that $$A \ne 0$$ and $$b \in \mathcal {K}$$. Consider the following optimization problem with two-block separable objective function and linear constraints

5 $$\begin{aligned}&\min _{x \in \mathcal {H}, z \in \mathcal {G}} f(x)+h_1(x)+g(z)+h_2(z) \quad \text {s.t.} \quad Ax+Bz=b. \end{aligned}$$

We allow the Lipschitz constant of the gradients of the functions $h_1$ and $h_2$ to be zero. In this case, the functions are affine.

The Lagrangian associated with the optimization problem (5) is defined by $L :\mathcal {H}\times \mathcal {G}\times \mathcal {K} \rightarrow \overline{\mathbb {R}},$

$$\begin{aligned} L(x,z,p)=f(x)+h_1(x)+g(z)+h_2(z)+\langle p, b-Ax-Bz \rangle . \end{aligned}$$

We say that $(x^*,z^*,p^*) \in \mathcal {H}\times \mathcal {G}\times \mathcal {K}$ is a saddle point of the Lagrangian *L*, if

$$\begin{aligned} (x^*,z^*,p) \le L(x^*,z^*,p^*) \le L(x,z,p^*) \quad \forall (x,z,p) \in \mathcal {H}\times \mathcal {G}\times \mathcal {K}. \end{aligned}$$

It is well known that $(x^*,z^*,p^*)$ is a saddle point of the Lagrangian *L* if and only if $(x^*,z^*)$ is an optimal solution of (5), $p^*$ is an optimal solution of its Fenchel dual problem

6 $$\begin{aligned} \sup _{\lambda \in \mathcal {K}}\{-(f^*\Box h_1^*)(A^*\lambda )-(g^*\Box h_2^*)(B^*\lambda )+\langle \lambda ,b\rangle \}, \end{aligned}$$

and the optimal objective values of (5) and (6) coincide. The existence of saddle points for *L* is guaranteed when (5) has an optimal solution and, for instance, the Attouch–Brézis-type condition

7 $$\begin{aligned} b\in {{\,\mathrm{sqri}\,}}(A({{\,\mathrm{dom}\,}}f)+B({{\,\mathrm{dom}\,}}g)) \end{aligned}$$

holds (see [12, Theorem 3.4]). In the finite-dimensional setting, this asks for the existence of $x \in {{\,\mathrm{ri}\,}}({{\,\mathrm{dom}\,}}f )$ and $z \in {{\,\mathrm{ri}\,}}({{\,\mathrm{dom}\,}}g)$ satisfying $Ax+Bz=b$ and coincides with the assumption used by Tseng [1].

The system of optimality conditions for the primal-dual pair of optimization problems (5)–(6) reads:

8 $$\begin{aligned} A^*p^*-\nabla h_1(x^*) \in \partial f(x^*), \ B^*p^*-\nabla h_2(z^*)\in \partial g(z^*) \ \text{ and } Ax^*+Bz^*=b. \end{aligned}$$

This means that if (5) has an optimal solution $(x^*,z^*)$ and a qualification condition, like for instance (7), is fulfilled, then there exists an optimal solution $p^*$ of (6) such that (8) holds; consequently, $(x^*,z^*,p^*)$ is a saddle point of the Lagrangian *L*. Conversely, if $(x^*,z^*,p^*)$ is a saddle point of the Lagrangian *L*, thus, $(x^*,z^*,p^*)$ satisfies relation (8), then $(x^*,z^*)$ is an optimal solution of (5) and $p^*$ is an optimal solution of (6).

### Remark 3.1

If $(x_1^*,z_1^*,p_1^*)$ and $(x_2^*,z_2^*,p_2^*)$ are two saddle points of the Lagrangian *L*, then $x_1^*=x_2^*$. This follows easily from (8), by using the strong monotonicity of $\partial f$ and the monotonicity of $\partial g$.

In the following, we formulate the Proximal Alternating Minimization Algorithm to solve (5). To this end, we modify Tseng’s AMA by evaluating in each of the two subproblems the functions $h_1$ and $h_2$ via gradient steps, respectively, and by introducing proximal terms defined through two sequences of positively semidefinite operators $(M_1^k)_{k \ge 0}$ and $(M_2^k)_{k \ge 0}$.

### Algorithm 3.1

*(Proximal AMA)* Let $(M_1^k)_{k \ge 0} \subseteq \mathcal {S}_+(\mathcal {H})$ and $(M_2^k)_{k \ge 0} \subseteq \mathcal {S}_+(\mathcal {G})$. Choose $(x^0,z^0,p^0) \!\!\in \mathcal {H}\times \mathcal {G}\times \mathcal {K}$ and a sequence of stepsizes $(c_k)_{k\ge 0} \subseteq (0,+\infty )$. For all $k\ge 0$, set:

9 $$\begin{aligned} x^{k+1}&={{\,\mathrm{argmin}\,}}_{x \in \mathcal {H}}\left\{ f(x)-\langle p^k,Ax\rangle +\,\langle x-x^{k}, \nabla h_1(x^{k}) \rangle +\frac{1}{2}\Vert x-x^{k}\Vert ^2_{M_1^{k}}\right\} \end{aligned}$$

10 $$\begin{aligned} z^{k+1}&\in {{\,\mathrm{argmin}\,}}_{z \in \mathcal {G}}\left\{ g(z)-\langle p^k,Bz\rangle \right. \nonumber \\&\quad \left. +\,\frac{c_k}{2} \Vert Ax^{k+1}+Bz-b\Vert ^2 +\langle z-z^k, \nabla h_2(z^k) \rangle +\frac{1}{2}\Vert z-z^k\Vert ^2_{M_2^{k}}\right\} \end{aligned}$$

11 $$\begin{aligned} p^{k+1}&=p^k+c_k(b-Ax^{k+1}-Bz^{k+1}). \end{aligned}$$

### Remark 3.2

The sequence $(z^k)_{k \ge 0}$ is uniquely determined if there exists $\alpha _k > 0$ such that $c_kB^*B+M_2^k \in \mathcal {P}_{\alpha _k}(\mathcal {G})$ for all $k \ge 0$. This actually ensures that the objective function in subproblem (10) is strongly convex.

### Remark 3.3

Let $k \ge 0$ be fixed and $M_2^k:=\frac{1}{\sigma _k}\text {Id}-c_kB^* B$, where $\sigma _k > 0$ and $\sigma _kc_k\Vert B\Vert ^2 \le 1$. Then $M_2^k$ is positively semidefinite, and the update of $z^{k+1}$ in the Proximal AMA method becomes a proximal step. This idea has been used in the past with the same purpose for different algorithms involving proximal steps; see, for instance, [7–9, 13–16]. Indeed, (10) holds if and only if

$$\begin{aligned}&0 \in \partial g(z^{k+1})+(c_kB^* B + M_2^k)z^{k+1}+c_kB^*(Ax^{k+1}-b)\\&\quad \quad -\,M_2^kz^{k}+\nabla h_2(z^{k})-B^* p^k\, \end{aligned}$$

or, equivalently,

$$\begin{aligned}&0 \in \partial g(z^{k+1})+\frac{1}{\sigma _k}z^{k+1} - \left( \frac{1}{\sigma _k}{{\,\mathrm{Id}\,}}-c_kB^* B\right) z^{k}\\&\quad \quad +\,\nabla h_2(z^{k})+ c_kB^*(Ax^{k+1}-b)-B^* p^k. \end{aligned}$$

But this is nothing else than

$$\begin{aligned} z^{k+1}= & {} {{\,\mathrm{argmin}\,}}_{z \in \mathcal {G}}\left\{ g(z)+\frac{1}{2\sigma _k}\left\| z-\left( z^{k}-\sigma _k\nabla h_2(z^{k}) \right. \right. \right. \\&\left. \left. \left. +\sigma _kc_kB^*(b-Ax^{k+1}-Bz^k)+\sigma _kB^* p^k \right) \right\| ^2 \right\} \\= & {} {{\,\mathrm{Prox}\,}}_{\sigma _k g}\left( z^{k}-\sigma _k\nabla h_2(z^{k})+\sigma _kc_kB^*(b-Ax^{k+1}-Bz^k)+\sigma _kB^* p^k \right) . \end{aligned}$$

The convergence of the Proximal AMA method is addressed in the next theorem.

### Theorem 3.1

In the setting of Problem 3.1, let the set of the saddle points of the Lagrangian *L* be nonempty. We assume that $M_1^k-\frac{L_1}{2}{{\,\mathrm{Id}\,}}\in \mathcal {S}_+(\mathcal {H}), M_1^k \succcurlyeq M_1^{k+1}$, $M_2^k-\frac{L_2}{2}{{\,\mathrm{Id}\,}}\in \mathcal {S}_+(\mathcal {G}), M_2^k \succcurlyeq M_2^{k+1}$ for all $k \ge 0$ and that $(c_k)_{k \ge 0}$ is a monotonically decreasing sequence satisfying

12 $$\begin{aligned} \epsilon \le c_k \le \frac{2\gamma }{\Vert A\Vert ^2}-\epsilon \quad \forall k \ge 0, \end{aligned}$$

where $0<\epsilon <\frac{\gamma }{\Vert A\Vert ^2}$. If one of the following assumptions:

(i) there exists $\alpha >0$ such that $M_2^k-\frac{L_2}{2}{{\,\mathrm{Id}\,}}\in \mathcal {P}_{\alpha }(\mathcal {G})$ for all $k \ge 0$;
(ii) there exists $\beta >0$ such that $B^*B\in \mathcal {P}_{\beta }(\mathcal {G})$;

holds true, then the sequence $(x^k,z^k,p^k)_{k \ge 0}$ generated by Algorithm 3.1 converges weakly to a saddle point of the Lagrangian *L*.

### Proof

Let $(x^*,z^*,p^*)$ be a fixed saddle point of the Lagrangian *L*. This means that it fulfils the system of optimality conditions

13 $$\begin{aligned}&A^*p^*-\nabla h_1(x^*) \in \partial f(x^*) \end{aligned}$$

14 $$\begin{aligned}&B^*p^*-\nabla h_2(z^*) \in \partial g(z^*) \end{aligned}$$

15 $$\begin{aligned}&Ax^*+Bz^* =b \end{aligned}$$

We start by proving that

$$\begin{aligned} \sum _{k\ge 0}\Vert x^{k+1}-x^*\Vert ^2< +\infty ,\quad \sum _{k\ge 0}\Vert Bz^{k+1}-Bz^*\Vert ^2< +\infty ,\quad \sum _{k\ge 0}\Vert z^{k+1}-z^k\Vert ^2_{M_2^{k}-\frac{L_2}{2}\text {Id}}< +\infty \end{aligned}$$

and that the sequences $(z^k)_{k \ge 0}$ and $(p^k)_{k \ge 0}$ are bounded.

Assume that $L_1 >0$ and $L_2 >0$. Let $k \ge 0$ be fixed. Writing the optimality conditions for subproblems (9) and (10), we obtain

16 $$\begin{aligned} A^*p^k-\nabla h_1(x^{k}) + M_1^k(x^{k}-x^{k+1})&\in \partial f(x^{k+1}) \end{aligned}$$

and

17 $$\begin{aligned} B^*p^k-\nabla h_2(z^{k}) + c_kB^*(-Ax^{k+1}-Bz^{k+1}+b)+M_2^k(z^{k}-z^{k+1})&\in \partial g(z^{k+1}), \end{aligned}$$

respectively. Combining (13)–(17) with the strong monotonicity of $\partial f$ and the monotonicity of $\partial g$, it yields

$$\begin{aligned}&\langle A^*(p^k-p^*)-\nabla h_1(x^{k})+\nabla h_1(x^*)\\&\quad +\,M_1^k(x^{k}-x^{k+1}), x^{k+1}-x^* \rangle \ge \gamma \Vert x^{k+1}-x^*\Vert ^2 \end{aligned}$$

and

$$\begin{aligned}&\langle B^*(p^k-p^*)-\nabla h_2(z^{k})+\nabla h_2(z^*)+ c_kB^*(-Ax^{k+1}-Bz^{k+1}+b)\\&\quad +\,M_2^k(z^{k}-z^{k+1}), z^{k+1}-z^* \rangle \ge 0, \end{aligned}$$

which after summation lead to

18 $$\begin{aligned}&\langle p^k-p^*,Ax^{k+1}-Ax^*\rangle + \langle p^k-p^*,Bz^{k+1}-Bz^*\rangle \nonumber \\&\quad + \langle c_k(-Ax^{k+1}-Bz^{k+1}+b),Bz^{k+1}-Bz^*\rangle \nonumber \\&\quad - \langle \nabla h_1(x^{k})-\nabla h_1(x^*), x^{k+1}-x^*\rangle -\langle \nabla h_2(z^{k})-\nabla h_2(z^*), z^{k+1}-z^*\rangle \nonumber \\&\quad + \langle M_1^k(x^{k}-x^{k+1}),x^{k+1}-x^*\rangle + \langle M_2^k(z^{k}-z^{k+1}),z^{k+1}-z^*\rangle \nonumber \\&\quad \ge \gamma \Vert x^{k+1}-x^*\Vert ^2. \end{aligned}$$

According to the Baillon–Haddad theorem (see [11, Corollary 18.16]), the gradients of $h_1$ and $h_2$ are $\frac{1}{L_1}$ and $\frac{1}{L_2}$-cocoercive, respectively; thus,

$$\begin{aligned} \langle \nabla h_1(x^*)-\nabla h_1(x^{k}), x^*-x^{k}\rangle \ge \frac{1}{L_1}\Vert \nabla h_1(x^*)-\nabla h_1(x^{k})\Vert ^2\\ \langle \nabla h_2(z^*)-\nabla h_2(z^{k}), z^*-z^{k}\rangle \ge \frac{1}{L_2}\Vert \nabla h_2(z^*)-\nabla h_2(z^{k})\Vert ^2. \end{aligned}$$

On the other hand, by taking into account (11) and (15), it holds

$$\begin{aligned}&\langle p^k-p^*,Ax^{k+1}-Ax^*\rangle + \langle p^k-p^*,Bz^{k+1}-Bz^*\rangle \\&\quad =\langle p^k-p^*, Ax^{k+1}+Bz^{k+1}-b \rangle = \frac{1}{c_k}\langle p^k-p^*, p^k-p^{k+1}\rangle \end{aligned}$$

By employing the last three relations in (18), it yields

$$\begin{aligned}&\frac{1}{c_k}\langle p^k-p^*, p^k-p^{k+1}\rangle + c_k\langle -Ax^{k+1}-Bz^{k+1}+b, Bz^{k+1}-Bz^* \rangle \\&\qquad + \langle M_1^k(x^{k}-x^{k+1}),x^{k+1}-x^*\rangle + \langle M_2^k(z^{k}-z^{k+1}),z^{k+1}-z^*\rangle \\&\qquad +\langle \nabla h_1(x^{*})-\nabla h_1(x^{k}), x^{k+1}-x^*\rangle +\langle \nabla h_1(x^{*})-\nabla h_1(x^{k}),x^*- x^{k}\rangle \\&\qquad -\frac{1}{L_1}\Vert \nabla h_1(x^*)-\nabla h_1(x^{k})\Vert ^2 +\langle \nabla h_2(z^{*})-\nabla h_2(z^{k}), z^{k+1}-z^*\rangle \\&\qquad +\langle \nabla h_2(z^{*})-\nabla h_2(z^{k}), z^*-z^{k}\rangle -\frac{1}{L_2}\Vert \nabla h_2(z^*)-\nabla h_2(z^{k})\Vert ^2\\&\quad \ge \gamma \Vert x^{k+1}-x^*\Vert ^2, \end{aligned}$$

which, after expressing the inner products by means of norms, becomes

$$\begin{aligned}&\frac{1}{2c_k}\left( \Vert p^k-p^*\Vert ^2+\Vert p^k-p^{k+1}\Vert ^2-\Vert p^{k+1}-p^*\Vert ^2\right) \\&\qquad +\frac{c_k}{2}\left( \Vert Ax^*-Ax^{k+1}\Vert ^2-\Vert b-Ax^{k+1}-Bz^{k+1}\Vert ^2-\Vert Ax^*+Bz^{k+1}-b\Vert ^2\right) \\&\qquad +\frac{1}{2}\left( \Vert x^{k}-x^*\Vert ^2_{M_1^k}-\Vert x^{k}-x^{k+1}\Vert ^2_{M_1^k}-\Vert x^{k+1}-x^*\Vert ^2_{M_1^k}\right) \\&\qquad +\frac{1}{2}\left( \Vert z^{k}-z^*\Vert ^2_{M_2^k}-\Vert z^{k}-z^{k+1}\Vert ^2_{M_2^k}-\Vert z^{k+1}-z^*\Vert ^2_{M_2^k}\right) \\&\qquad +\langle \nabla h_1(x^{*})-\nabla h_1(x^{k}), x^{k+1}-x^{k}\rangle -\frac{1}{L_1}\Vert \nabla h_1(x^*)-\nabla h_1(x^{k})\Vert ^2 \\&\qquad +\langle \nabla h_2(z^{*})-\nabla h_2(z^{k}), z^{k+1}-z^{k}\rangle -\frac{1}{L_2}\Vert \nabla h_2(z^*)-\nabla h_2(z^{k})\Vert ^2 \\&\quad \ge \gamma \Vert x^{k+1}-x^*\Vert ^2. \end{aligned}$$

Using again (11), inequality $\Vert Ax^*-Ax^{k+1}\Vert ^2\le \Vert A\Vert ^2\Vert x^*-x^{k+1}\Vert ^2$ and the following expressions

$$\begin{aligned}&\langle \nabla h_1(x^*)-\nabla h_1(x^{k}), x^{k+1}-x^{k}\rangle - \frac{1}{L_1}\Vert \nabla h_1(x^*)-\nabla h_1(x^{k})\Vert ^2\\&\quad =-L_1\left\| \frac{1}{L_1}(\nabla h_1(x^*)-\nabla h_1(x^{k}))+\frac{1}{2}(x^{k}-x^{k+1}) \right\| ^2+\frac{L_1}{4}\Vert x^{k}-x^{k+1}\Vert ^2, \end{aligned}$$

and

$$\begin{aligned}&\langle \nabla h_2(x^*)-\nabla h_2(z^{k}), z^{k+1}-z^{k}\rangle - \frac{1}{L_2}\Vert \nabla h_2(z^*)-\nabla h_2(z^{k})\Vert ^2\\&\quad =-L_2\left\| \frac{1}{L_2}(\nabla h_2(z^*)-\nabla h_2(z^{k}))+\frac{1}{2}(z^{k}-z^{k+1})\right\| ^2+\frac{L_2}{4}\Vert z^{k}-z^{k+1}\Vert ^2, \end{aligned}$$

it yields

$$\begin{aligned}&\frac{1}{2}\Vert x^{k+1}-x^*\Vert ^2_{M_1^k}+\frac{1}{2c_k}\Vert p^{k+1}-p^*\Vert ^2+\frac{1}{2}\Vert z^{k+1}-z^*\Vert ^2_{M_2^k}\\&\quad \le \quad \frac{1}{2}\Vert x^{k}-x^*\Vert ^2_{M_1^k}+\frac{1}{2c_k}\Vert p^k-p^*\Vert ^2+\frac{1}{2}\Vert z^{k}-z^*\Vert ^2_{M_2^k}-\frac{c_k}{2}\Vert Ax^*+Bz^{k+1}-b\Vert ^2\\&\qquad -\frac{1}{2}\Vert z^{k}-z^{k+1}\Vert ^2_{M_2^k}-\left( \gamma -\frac{c_k}{2}\Vert A\Vert ^2\right) \Vert x^{k+1}-x^*\Vert ^2-\frac{1}{2}\Vert x^{k}-x^{k+1}\Vert ^2_{M_1^k}\\&\qquad -L_1\left\| \frac{1}{L_1}(\nabla h_1(x^*)-\nabla h_1(x^{k}))+\frac{1}{2}(x^{k}-x^{k+1})\right\| ^2+\frac{L_1}{4}\Vert x^{k}-x^{k+1}\Vert ^2\\&\qquad -L_2\left\| \frac{1}{L_2}(\nabla h_2(z^*)-\nabla h_2(z^{k}))+\frac{1}{2}(z^{k}-z^{k+1})\right\| ^2+\frac{L_2}{4}\Vert z^{k}-z^{k+1}\Vert ^2. \end{aligned}$$

Finally, by using the monotonicity of $(M_1^k)_{k\ge 0}, (M_2^k)_{k\ge 0}$ and $(c_k)_{k\ge 0}$, we obtain

19 $$\begin{aligned}&c_{k+1}\Vert x^{k+1}-x^*\Vert ^2_{M_1^{k+1}}+\Vert p^{k+1}-p^*\Vert ^2+c_{k+1}\Vert z^{k+1}-z^*\Vert ^2_{M_2^{k+1}}\nonumber \\&\quad \le c_{k}\Vert x^{k}-x^*\Vert ^2_{M_1^{k}}+\Vert p^k-p^*\Vert ^2+c_k\Vert z^{k}-z^*\Vert ^2_{M_2^k} - R_k, \end{aligned}$$

where

$$\begin{aligned} R_k&:= \ c_k\left( 2\gamma -c_k\Vert A\Vert ^2\right) \Vert x^{k+1}-x^*\Vert ^2 +c_k^2\Vert Bz^{k+1}-Bz^*\Vert ^2 \\&\quad +\ c_k\Vert z^{k}-z^{k+1}\Vert ^2_{M_2^k-\frac{L_2}{2}{{\,\mathrm{Id}\,}}}+c_k\Vert x^{k}-x^{k+1}\Vert ^2_{M_1^k-\frac{L_1}{2}{{\,\mathrm{Id}\,}}} \\&\quad +\ 2c_kL_1\left\| \frac{1}{L_1}(\nabla h_1(x^*)-\nabla h_1(x^{k}))+\frac{1}{2}(x^{k}-x^{k+1})\right\| ^2 \\&\quad +\ 2c_kL_2\left\| \frac{1}{L_2}(\nabla h_2(z^*)-\nabla h_2(z^{k}))+\frac{1}{2}(z^{k}-z^{k+1})\right\| ^2. \end{aligned}$$

If $L_1 =0$ (and, consequently, $\nabla h_1$ is constant) and $L_2 >0$, then, by using the same arguments, we obtain again (19), but with

$$\begin{aligned} R_k&:= \ c_k\left( 2\gamma -c_k\Vert A\Vert ^2\right) \Vert x^{k+1}-x^*\Vert ^2 +c_k^2\Vert Bz^{k+1}-Bz^*\Vert ^2 \\&\quad +\ c_k\Vert z^{k}-z^{k+1}\Vert ^2_{M_2^k-\frac{L_2}{2}{{\,\mathrm{Id}\,}}}+c_k\Vert x^{k}-x^{k+1}\Vert ^2_{M_1^k}\\&\quad +\ 2c_kL_2\left\| \frac{1}{L_2}(\nabla h_2(z^*)-\nabla h_2(z^{k}))+\frac{1}{2}(z^{k}-z^{k+1})\right\| ^2. \end{aligned}$$

If $L_2 = 0$ (and, consequently, $\nabla h_2$ is constant) and $L_2 >0$, then, by using the same arguments, we obtain again (19), but with

$$\begin{aligned} R_k&:= \ c_k\left( 2\gamma -c_k\Vert A\Vert ^2\right) \Vert x^{k+1}-x^*\Vert ^2 +c_k^2\Vert Bz^{k+1}-Bz^*\Vert ^2 \\&\quad +\ c_k\Vert z^{k}-z^{k+1}\Vert ^2_{M_2^k}+c_k\Vert x^{k}-x^{k+1}\Vert ^2_{M_1^k-\frac{L_1}{2}{{\,\mathrm{Id}\,}}} \\&\quad + \ 2c_kL_1\left\| \frac{1}{L_1}(\nabla h_1(x^*)-\nabla h_1(x^{k}))+\frac{1}{2}(x^{k}-x^{k+1})\right\| ^2. \end{aligned}$$

Relation (19) follows even if $L_1=L_2=0$, but with

$$\begin{aligned} R_k&:= \ c_k\left( 2\gamma -c_k\Vert A\Vert ^2\right) \Vert x^{k+1}-x^*\Vert ^2 +c_k^2\Vert Bz^{k+1}-Bz^*\Vert ^2 \\&\quad +\, c_k\Vert z^{k}-z^{k+1}\Vert ^2_{M_2^k}+c_k\Vert x^{k}-x^{k+1}\Vert ^2_{M_1^k}. \end{aligned}$$

Notice that, due to $M_1^{k}-\frac{L_1}{2}{{\,\mathrm{Id}\,}}\in \mathcal {S}_+(\mathcal {H})$ and $M_2^{k}-\frac{L_2}{2}{{\,\mathrm{Id}\,}}\in \mathcal {S}_+(\mathcal {G})$, all summands in $R_k$ are nonnegative.

Let be $N \ge 0$ fixed. By summing the inequality in (19) for $k=0, \ldots , N$ and using telescoping arguments, we obtain

$$\begin{aligned}&c_{N+1}\Vert x^{N+1}-x^*\Vert ^2_{M_1^{N+1}}+\Vert p^{N+1}-p^*\Vert ^2+c_N\Vert z^{N+1}-z^*\Vert ^2_{M_2^{N+1}}\\&\quad \le c_{0}\Vert x^{0}-x^*\Vert ^2_{M_1^{0}}+\Vert p^0-p^*\Vert ^2+c_0\Vert z^0-z^*\Vert _{M_2^0}-\sum _{k=0}^{N} R_k. \end{aligned}$$

On the other hand, from (19) we also obtain that

20 $$\begin{aligned} \exists \lim _{k \rightarrow \infty } \left( c_{k}\Vert x^{k}-x^*\Vert ^2_{M_1^{k}}+\Vert p^k-p^*\Vert ^2+c_k\Vert z^{k}-z^*\Vert ^2_{M_2^{k}}\right) , \end{aligned}$$

thus $(p^k)_{k \ge 0}$ is bounded, and $\sum _{k \ge 0} R_k < + \infty$.

Taking (12) into account, we have $c_k(2\gamma -c_k\Vert A\Vert ^2)\ge \varepsilon ^2\Vert A\Vert ^2$ for all $k \ge 0$. Therefore,

21 $$\begin{aligned} \sum _{k\ge 0}\Vert x^{k+1}-x^*\Vert ^2< +\infty , \quad \sum _{k\ge 0}\Vert Bz^{k+1}-Bz^*\Vert ^2< +\infty \end{aligned}$$

and

22 $$\begin{aligned} \sum _{k\ge 0}\Vert z^{k+1}-z^{k}\Vert ^2_{M_2^{k}-\frac{L_2}{2}{{\,\mathrm{Id}\,}}}< +\infty . \end{aligned}$$

From here, we obtain

23 $$\begin{aligned} x^k \rightarrow x^*, \quad Bz^k \rightarrow Bz^* \ (k \rightarrow +\infty ), \end{aligned}$$

which, by using (11) and (15), lead to

24 $$\begin{aligned} p^{k}-p^{k+1} \rightarrow 0 \ (k \rightarrow +\infty ). \end{aligned}$$

Taking into account the monotonicity properties of $(c_k)_{k\ge 0}$ and $(M_1^k)_{k\ge 0}$, a direct implication of (20) and (23) is

25 $$\begin{aligned} \exists \lim _{k \rightarrow \infty } \left( \Vert p^k-p^*\Vert ^2+c_k\Vert z^{k}-z^*\Vert ^2_{M_2^{k}}\right) . \end{aligned}$$

Suppose that assumption (i) holds true, namely that there exists $\alpha > 0$ such that $M_2^k-\frac{L_2}{2}{{\,\mathrm{Id}\,}}\in \mathcal {P}_\alpha (\mathcal {G})$ for all $k \ge 0$. From (25), it follows that $(z^k)_{k \ge 0}$ is bounded, while (22) ensures that

26 $$\begin{aligned} z^{k+1}-z^{k} \rightarrow 0 \ (k \rightarrow +\infty ). \end{aligned}$$

In the following, let us prove that each weak sequential cluster point of $(x^k,z^k,p^k)_{k\ge 0}$ (notice that the sequence is bounded) is a saddle point of *L*. Let be $({\bar{z}},{\bar{p}}) \in \mathcal {G}\times \mathcal {K}$ such that the subsequence $(x^{k_j}, z^{k_j}, p^{k_j})_{j\ge 0}$ converges weakly to $(x^*,{\bar{z}},{\bar{p}})$ as $j\rightarrow +\infty$. From (16), we have

$$\begin{aligned} A^*p^{k_j}-\nabla h_1(x^{k_j}) + M_1^{k_j}(x^{k_j}-x^{k_j+1}) \in \partial f(x^{k_j+1}) \quad \forall j \ge 1. \end{aligned}$$

Due to the fact that $x^{k_j}$ converges strongly to $x^*$ and $p^{k_j}$ converges weakly to a ${\bar{p}}$ as $j \rightarrow +\infty$, using the continuity of $\nabla h_1$ and the fact that the graph of the convex subdifferential of *f* is sequentially closed in the strong-weak topology (see [11, Proposition 20.33]), it follows

$$\begin{aligned} A^*{\bar{p}}-\nabla h_1(x^*) \in \partial f(x^*). \end{aligned}$$

From (17), we have for all $j \ge 0$

$$\begin{aligned}&B^*p^{k_j}-\nabla h_2(z^{k_j}) + c_{k_j}B^*(-Ax^{k_j+1}-Bz^{k_j+1}+b)\\&\quad +\,M_2^{k_j}(z^{k_j}-z^{k_j+1}) \in \partial g(z^{k_j+1}), \end{aligned}$$

which is equivalent to

$$\begin{aligned}&B^*p^{k_j}+\nabla h_2(z^{k_j+1})-\nabla h_2(z^{k_j}) + c_{k_j}B^*(-Ax^{k_j+1}-Bz^{k_j+1}+b)\nonumber \\&\quad +\,M_2^{k_j}(z^{k_j}-z^{k_j+1})\in \partial (g+h_2)(z^{k_j+1}) \end{aligned}$$

and further to

27 $$\begin{aligned}&z^{k_j+1} \in \partial (g+h_2)^*\Big (B^*p^{k_j} + \nabla h_2(z^{k_j+1})-\nabla h_2(z^{k_j}) \nonumber \\&\quad +\, c_{k_j}B^*(-Ax^{k_j+1}-Bz^{k_j+1}+b)+M_2^{k_j}(z^{k_j}-z^{k_j+1})\Big ). \end{aligned}$$

By denoting for all $j \ge 0$

$$\begin{aligned} v^j&:=z^{k_j+1}, u^j : =p^{k_j},\\ w^j&:=\nabla h_2(z^{k_j+1})-\nabla h_2(z^{k_j})\\&\quad +\,c_{k_j}B^*(-Ax^{k_j+1}-Bz^{k_j+1}+b)+M_2^{k_j}(z^{k_j}-z^{k_j+1}), \end{aligned}$$

(27) reads

$$\begin{aligned} v^j \in \partial (g+h_2)^*(B^*u^j+w^j) \quad \forall j \ge 0. \end{aligned}$$

According to (26), we have $v^j \rightharpoonup {\bar{z}}, u^j \rightharpoonup {\bar{p}}$ as $j \rightarrow +\infty$; thus, by taking into account (23), $Bv^j \rightarrow B {{\bar{z}}} = Bz^*$ as $j \rightarrow +\infty$. Combining (29) with the Lipschitz continuity of $\nabla h_2$, (24), (26) and (11), one can easily see that $w^j \rightarrow 0$ as $j \rightarrow +\infty$. Due to the monotonicity of the subdifferential, we have that for all (*u*, *v*) in the graph of $\partial (g+h_2)^*$ and for all $j\ge 0$

$$\begin{aligned} \langle Bv^j-Bv,u^j \rangle + \langle v^j-v,w^j-u\rangle \ge 0. \end{aligned}$$

We let *j* converge to $+\infty$ and receive

$$\begin{aligned} \langle {\bar{z}}-v,B^*{\bar{p}} -u \rangle \ge 0 \ \ \forall (u,v) \text{ in } \text{ the } \text{ graph } \text{ of } \partial (g+h_2)^*. \end{aligned}$$

The maximal monotonicity of the convex subdifferential of $(g+h_2)^*$ ensures that ${\bar{z}} \in \partial (g+h_2)^*(B^*{\bar{p}})$, which is the same as $B^*{\bar{p}} \in \partial (g+h_2)({\bar{z}})$. In other words, $B^*{\bar{p}}-\nabla h_2({\bar{z}}) \in \partial g({\bar{z}})$. Finally, by combining (11) and (24), the equality $Ax^* + B{{\bar{z}}} = b$ follows. In conclusion, $(x^*, {{\overline{z}}},{\bar{p}})$ is a saddle point of the Lagrangian *L*.

In the following, we show that sequence $(x^k,z^k,p^k)_{k \ge 0}$ converges weakly. To this end, we consider two sequential cluster points $(x^*,z_1,p_1)$ and $(x^*,z_2,p_2)$. Consequently, there exists $(k_s)_{s \ge 0}$, $k_s \rightarrow + \infty$ as $s \rightarrow + \infty$, such that the subsequence $(x^{k_s}, z^{k_s}, p^{k_s})_{s \ge 0}$ converges weakly to $(x^*,z_1,p_1)$ as $s \rightarrow + \infty$. Furthermore, there exists $(k_t)_{t \ge 0}$, $k_t \rightarrow + \infty$ as $t \rightarrow + \infty$, such that that a subsequence $(x^{k_t}, z^{k_t}, p^{k_t})_{t \ge 0}$ converges weakly to $(x^*,z_2,p_2)$ as $t \rightarrow + \infty$. As seen before, $(x^*,z_1,p_1)$ and $(x^*,z_2,p_2)$ are both saddle points of the Lagrangian *L*.

From (25), which is fulfilled for every saddle point of the Lagrangian *L*, we obtain

28 $$\begin{aligned} \exists \lim _{k \rightarrow + \infty }(\Vert p^k\!-\!p_1\Vert ^2-\Vert p^k-p_2\Vert ^2\!+\!c_k\Vert z^{k}-z_1\Vert ^2_{M_2^k}\!-\!c_k\Vert z^{k}\!-\!z_2\Vert ^2_{M_2^k}):\!=\!T. \end{aligned}$$

For all $k \ge 0$, we have

$$\begin{aligned}&\Vert p^k-p_1\Vert ^2-\Vert p^k-p_2\Vert ^2+c_k\Vert z^{k}-z_1\Vert ^2_{M_2^k}-c_k\Vert z^{k}-z_2\Vert ^2_{M_2^k}\\&\quad =\Vert p_2-p_1\Vert ^2+2\langle p_k-p_2, p_2-p_1 \rangle + c_k\Vert z_2-z_1\Vert _{M_2^k}^2\\&\qquad +\,2c_k\langle z_{k}-z_2, z_2-z_1 \rangle _{M_2^k}. \end{aligned}$$

Since $M_2^k \ge \left( \alpha + \frac{L_2}{2} \right) {{\,\mathrm{Id}\,}}$ for all $k \ge 0$ and $(M_2^k)_{k \ge 0}$ is a nonincreasing sequence of symmetric operators in the sense of the Loewner partial ordering, there exists a symmetric operator $M \ge \left( \alpha + \frac{L_2}{2} \right) {{\,\mathrm{Id}\,}}$ such that $(M_2^k)_{k \ge 0}$ converges pointwise to *M* in the strong topology as $k \rightarrow +\infty$ (see [17, Lemma 2.3]). Furthermore, let $c:=\lim _{k \rightarrow +\infty } c_k >0$. Taking the limits in (28) along the subsequences $(k_s)_{s \ge 0}$ and $(k_t)_{t \ge 0}$, it yields

$$\begin{aligned} T=-\Vert p_2-p_1\Vert ^2-c\Vert z_2-z_1\Vert ^2_M =\Vert p_2-p_1\Vert ^2+c\Vert z_2-z_1\Vert ^2_M, \end{aligned}$$

thus

$$\begin{aligned} \Vert p_2-p_1\Vert ^2+c\Vert z_2-z_1\Vert ^2_M=0. \end{aligned}$$

It follows that $p_1=p_2$ and $z_1=z_2$; thus, $(x^k, z^k, p^k)_{k\ge 0}$ converges weakly to a saddle point of the Lagrangian *L*.

Assume now that condition (ii) holds, namely that there exists $\beta > 0$ such that $B^*B \in \mathcal {P}_\beta (\mathcal {H})$. Then $\beta \Vert z_1-z_2\Vert ^2 \le \Vert Bz_1-Bz_2\Vert ^2$ for all $z_1, z_2 \in \mathcal {G}$, which means that, if $(x_1^*,z_1^*,p_1^*)$ and $(x_2^*,z_2^*,p_2^*)$ are two saddle points of the Lagrangian *L*, then $x_1^*=x_2^*$ and $z_1^*=z_2^*$.

For the saddle point $(x^*, z^*, p^*)$ of the Lagrangian *L*, we fixed at the beginning of the proof and the generated sequence $(x^k, z^k, p^k)_{k \ge 0}$ we receive because of (23) that

29 $$\begin{aligned} x^k \rightarrow x^*, \quad z^k \rightarrow z^*, \quad p^{k} - p^{k+1} \rightarrow 0 \ (k \rightarrow +\infty ). \end{aligned}$$

Moreover,

$$\begin{aligned} \exists \lim _{k \rightarrow \infty } \Vert p^k-p^*\Vert ^2. \end{aligned}$$

The remainder of the proof follows in analogy to the one given under assumption (i). $\square$

If $h_1=0$ and $h_2=0$, and $M_1^k=0$ and $M_2^k = 0$ for all $k \ge 0$, then the Proximal AMA method becomes the AMA method as it has been proposed by Tseng [1]. According to Theorem 3.1 (for $L_1=L_2=0$), the generated sequence converges weakly to a saddle point of the Lagrangian, if there exists $\beta >0$ such that $B^*B\in \mathcal {P}_{\beta }(\mathcal {G})$. In finite-dimensional spaces, this condition reduces to the assumption that *B* is injective.

## Numerical Experiments

In this section, we compare the numerical performances of AMA and Proximal AMA on two applications in image processing and machine learning. The numerical experiments were performed on a computer with an Intel Core i5-3470 CPU and 8 GB DDR3 RAM.

### Image Denoising and Deblurring

We addressed an image denoising and deblurring problem formulated as a nonsmooth convex optimization problem (see [18]–[20]])

30 $$\begin{aligned} \inf _{x \in \mathbb {R}^n} \left\{ \frac{1}{2}\Vert Ax-b\Vert ^2 + \lambda \text {TV}(x)\right\} , \end{aligned}$$

where $A \in \mathbb {R}^{n\times n}$ represents a blur operator, $b \in \mathbb {R}^n$ is a given blurred and noisy image, $\lambda >0$ is a regularization parameter and $\text {TV}:\mathbb {R}^n\rightarrow \mathbb {R}$ is a discrete total variation functional. The vector $x \in \mathbb {R}^n$ is the vectorized image $X \in \mathbb {R}^{M \times N}$, where $n=MN$ and $x_{i,j} := X_{i,j}$ stand for the normalized value of the pixel in the *i*-th row and the *j*-th column, for $1 \le i \le M, 1 \le j \le N$.

Two choices have been considered for the discrete total variation, namely the isotropic total variation $\text {TV}_{\text {iso}}:\mathbb {R}^n\rightarrow \mathbb {R},$

$$\begin{aligned} \text {TV}_{\text {iso}}(x)&=\sum _{i=1}^{M-1}\sum _{j=1}^{N-1}\sqrt{(x_{i+1,j}-x_{i,j})^2+(x_{i,j+1}-x_{i,j})^2}\\&\quad +\,\sum _{i=1}^{M-1}|x_{i+1,N}-x_{i,j}|+\sum _{j=1}^{N-1}|x_{M,j+1}-x_{M,j}|, \end{aligned}$$

and the anisotropic total variation $\text {TV}_{\text {aniso}}:\mathbb {R}^n\rightarrow \mathbb {R},$

$$\begin{aligned} \text {TV}_{\text {aniso}}(x)&=\sum _{i=1}^{M-1}\sum _{j=1}^{N-1}|x_{i+1,j}-x_{i,j}|+|x_{i,j+1}-x_{i,j}| \\&\quad +\,\sum _{i=1}^{M-1}|x_{i+1,N}-x_{i,j}|+\sum _{j=1}^{N-1}|x_{M,j+1}-x_{M,j}|. \end{aligned}$$

Consider the linear operator $L:\mathbb {R}^n\rightarrow \mathbb {R}^n \times \mathbb {R}^n, x_{i,j} \mapsto \left( L_1x_{i,j},L_2x_{i,j}\right)$, where

$$\begin{aligned} L_1x_{i,j}={\left\{ \begin{array}{ll} x_{i+1,j}-x_{i,j}, &{}\quad \text {if } i<M\\ 0, &{}\quad \text {if } i=M \end{array}\right. } \text { and } L_2x_{i,j}={\left\{ \begin{array}{ll} x_{i,j+1}-x_{i,j}, &{}\quad \text {if } j<N\\ 0, &{}\quad \text {if } j=N \end{array}\right. } \end{aligned}$$

One can easily see that $\Vert L\Vert ^2\le 8$. The optimization problem (30) can be written as

31 $$\begin{aligned} \inf _{x \in \mathbb {R}^n}\left\{ f(Ax)+g(Lx)\right\} , \end{aligned}$$

where $f:\mathbb {R}^n \rightarrow \mathbb {R}, f(x)=\frac{1}{2}\Vert x-b\Vert ^2$, and $g:\mathbb {R}^n \times \mathbb {R}^n \rightarrow \mathbb {R}$ is defined by $g(y,z)=\lambda \Vert (y,z)\Vert _1$ for the anisotropic total variation, and by $g(y,z)=\lambda \Vert (y,z)\Vert _{\times }:=\lambda \sum _{i=1}^M\sum _{j=1}^N\sqrt{y_{i,j}^2+z_{i,j}^2}$ for the isotropic total variation.

We solved the Fenchel dual problem of (31) by AMA and Proximal AMA and determined in this way an optimal solution of the primal problem, too. The reason for this strategy was that the Fenchel dual problem of (31) is a convex optimization problem with two-block separable linear constraints and objective function.

Indeed, the Fenchel dual problem of (31) reads (see [11, 12])

32 $$\begin{aligned} \inf _{p \in \mathbb {R}^n, q \in \mathbb {R}^n \times \mathbb {R}^n}\left\{ f^*(p) + g^*(q)\right\} , \text { s.t. }A^* p+L^* q=0. \end{aligned}$$

Since *f* and *g* have full domains, strong duality for (31)–(32) holds.

As $f^*(p)=\frac{1}{2}\Vert p\Vert ^2+\langle p, b\rangle$ for all $p \in \mathbb {R}^n$, $f^*$ is 1-strongly convex. We chose $M_1^k = 0$ and $M_2^k=\frac{1}{\sigma _k}\text {I}-c_kL^* L$ (see Remark 3.3) and obtained for Proximal AMA the iterative scheme which reads for every $k \ge 0$ :

$$\begin{aligned} p^{k+1}&=Ax^k-b\\ q^{k+1}&=\text {Prox}_{\sigma _kg^*}\left( q^{k}+\sigma _kc_kL(-A^* p^{k+1} - L^*q^k)+\sigma _kL(x^k)\right) \\ x^{k+1}&=x^k+c_k(-A^* p^{k+1}-L^* q^{k+1}). \end{aligned}$$

In the case of the anisotropic total variation, the conjugate of *g* is the indicator function of the set $[-\lambda ,\lambda ]^n\times [-\lambda ,\lambda ]^n$; thus, $\text {Prox}_{\sigma _kg^*}$ is the projection operator $\mathcal {P}_{[-\lambda ,\lambda ]^n\times [-\lambda ,\lambda ]^n}$ on the set $[-\lambda ,\lambda ]^n\times [-\lambda ,\lambda ]^n$. The iterative scheme reads for all $k \ge 0$:

$$\begin{aligned} p^{k+1}&=Ax^k-b\\ (q_1^{k+1},q_2^{k+1})&=\mathcal {P}_{[-\lambda ,\lambda ]^n\times [-\lambda ,\lambda ]^n}\left( (q_1^{k},q_2^{k})\right. \\&\quad \left. +\,c_k\sigma _k(-LA^* p^{k+1} - LL^* (q_1^{k},q_2^{k}))+\sigma _kLx^k\right) \\ x^{k+1}&=x^k+c_k\left( -A^* p^{k+1}-L^*(q_1^{k+1},q_2^{k+1})\right) . \end{aligned}$$

In the case of the isotropic total variation, the conjugate of *g* is the indicator function of the set $S:=\left\{ (v,w)\in \mathbb {R}^n\times \mathbb {R}^n:\max _{1\le i\le n}\sqrt{v_i^2+w_i^2}\le \lambda \right\}$; thus, $\text {Prox}_{\sigma _kg^*}$ is the projection operator $P_S : \mathbb {R}^n \times \mathbb {R}^n \rightarrow S$ on *S*, defined as

$$\begin{aligned} (v_i,w_i) \mapsto \lambda \frac{(v_i,w_i)}{\max \left\{ \lambda , \sqrt{v_i^2+w_i^2}\right\} }, \quad i=1,\ldots ,n. \end{aligned}$$

The iterative scheme reads for all $k \ge 0$:

$$\begin{aligned} p^{k+1}&=Ax^k-b\\ (q_1^{k+1},q_2^{k+1})&=P_S\left( (q_1^{k},q_2^{k})+c_k\sigma _k(-LA^* p^{k+1} - LL^* (q_1^{k},q_2^{k}))+\sigma _kLx^k\right) \\ x^{k+1}&=x^k+c_k\left( -A^* p^{k+1}-L^*(q_1^{k+1},q_2^{k+1})\right) . \end{aligned}$$

We compared the Proximal AMA method with Tseng’s AMA method. While in Proximal AMA a closed formula is available for the computation of $(q_1^{k+1},q_2^{k+1})_{k \ge 0}$, in AMA we solved the resulting optimization subproblem

$$\begin{aligned} (q_1^{k+1},q_2^{k+1})= & {} {{\,\mathrm{argmin}\,}}_{q_1,q_2}\left\{ g^*(q_1,q_2)-\langle x^{k+1},L^*(q_1,q_2)\right. \rangle \\&\quad \left. +\,\frac{1}{2}c_k\Vert A^*p^{k+1}+L^*(q_1,q_2)\Vert ^2\right\} \end{aligned}$$

in every iteration $k \ge 0$ by making some steps of the FISTA method [2].

We used in our experiments a Gaussian blur of size $9 \times 9$ and standard deviation 4, which led to an operator *A* with $\Vert A\Vert ^2=1$ and $A^*=A$. Furthermore, we added Gaussian white noise with standard deviation $10^{-3}$. We used for both algorithms a constant sequence of stepsizes $c_k=2 -10^{-7}$ for all $k \ge 0$. One can notice that $(c_k)_{k \ge 0}$ fulfils (12). For Proximal AMA, we considered $\sigma _k=\frac{1}{8.00001 \cdot c_k}$ for all $k \ge 0$, which ensured that every matrix $M_2^k=\frac{1}{\sigma _k}\text {I}-c_kL^* L$ is positively definite for all $k \ge 0$. This is actually the case, if $\sigma _kc_k\Vert L\Vert ^2<1$ for all $k \ge 0$. In other words, assumption (i) in Theorem 3.1 was verified.

**Fig. 1:**
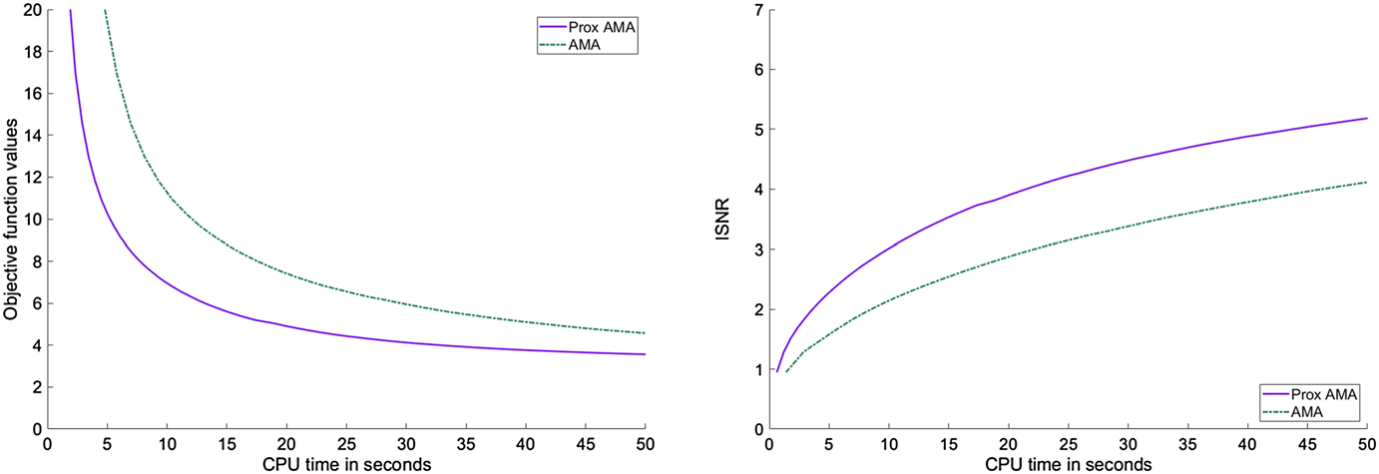
Objective function values and the ISNR values for the anisotropic TV and $\lambda =5\cdot 10^{-5}$

**Fig. 2:**
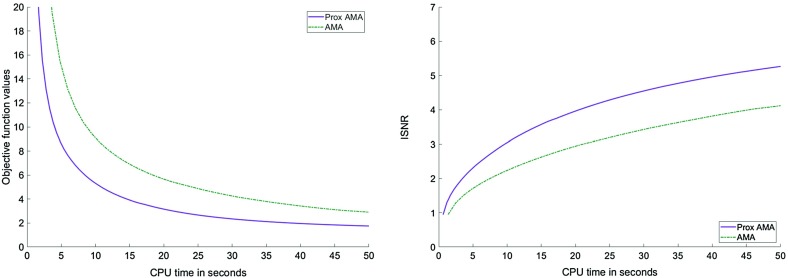
Objective function values and the ISNR values for the anisotropic TV and $\lambda =10^{-5}$

**Fig. 3:**
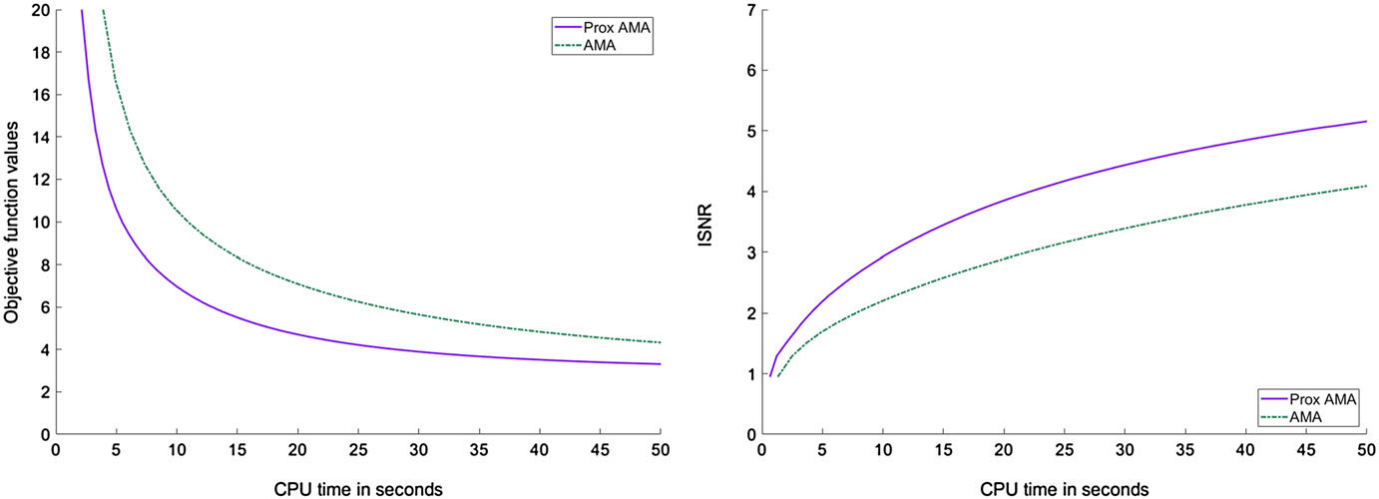
Objective function values and the ISNR values for the isotropic TV and $\lambda =5\cdot 10^{-5}$

**Fig. 4:**
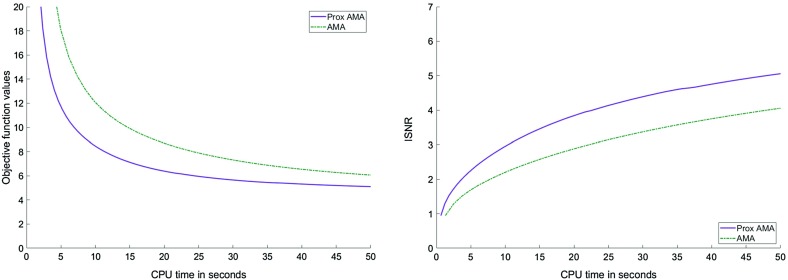
Objective function values and the ISNR values for the isotropic TV and $\lambda =10^{-4}$

**Fig. 5:**
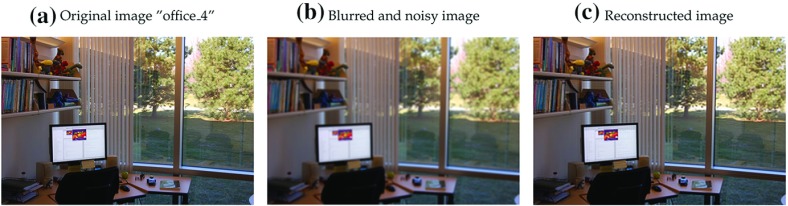
Original image, the blurred and noisy image and the reconstructed image after 50 s cpu time

In Figs. 1, 2, 3 and 4, we show how Proximal AMA and AMA perform when reconstructing the blurred and noisy coloured MATLAB test image “office_ 4” of $600 \times 903$ pixels (see Fig. 5) for different choices for the regularization parameter $\lambda$ and by considering both the anisotropic and isotropic total variation as regularization functionals. In all considered instances that Proximal AMA outperformed AMA from the point of view of both the convergence behaviour of the sequence of the function values and of the sequence of ISNR (Improvement in signal-to-noise ratio) values. An explanation could be that the number of iterations Proximal AMA makes in a certain amount of time is more than double the number of outer iterations performed by AMA.

### Kernel-Based Machine Learning

In this subsection, we will describe the numerical experiments we carried out in the context of classifying images via support vector machines.

**Fig. 6:**
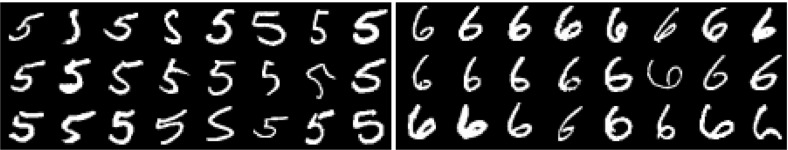
A sample of images belonging to the classes $+1$ and $-1$, respectively

The given data set consisting of 5570 training images and 1850 test images of size $28 \times 28$ was taken from http://www.cs.nyu.edu/~roweis/data.html. The problem we considered was to determine a decision function based on a pool of handwritten digits showing either the number five or the number six, labelled by $+1$ and $-1$, respectively (see Fig. 6). To evaluate the quality of the decision function, we computed the percentage of misclassified images of the test data set.

In order to describe the approach we used, we denote by

$$\begin{aligned} \mathcal {Z}=\{(X_1,Y_1),\ldots ,(X_n,Y_n)\}\subseteq \mathbb {R}^d \times \{+1,-1\}, \end{aligned}$$

the given training data set. The decision functional $\mathtt{{f}}$ was assumed to be an element of the Reproducing Kernel Hilbert Space (RHKS) $\mathcal {H}_\kappa$, induced by the symmetric and finitely positive definite Gaussian kernel function

$$\begin{aligned} \kappa : \mathbb {R}^d \times \mathbb {R}^d \rightarrow \mathbb {R}, \ \kappa (x,y) = \exp \left( -\frac{\left\| x-y \right\| ^2}{2 \sigma ^2} \right) . \end{aligned}$$

By $K\in \mathbb {R}^{n\times n}$, we denoted the Gram matrix with respect to the training data set $\mathcal {Z}$, namely the symmetric and positive definite matrix with entries $K_{ij}=\kappa (X_i,X_j)$ for $i,j=1,\ldots ,n$. To penalize the deviation between the predicted value $\mathtt{{f}}(x)$ and the true value $y \in \{+1,-1\}$, we used the hinge loss functional $(x,y) \mapsto \max \{1-xy,0\}$.

According to the representer theorem, the decision function $\mathtt{{f}}$ can be expressed as a kernel expansion in terms of the training data; in other words, $\mathtt{{f}}(\cdot ) = \sum _{i=1}^n x_i \kappa (\cdot , X_i)$, where $x=(x_1,\ldots ,x_n) \in \mathbb {R}^n$ is the optimal solution of the optimization problem

33 $$\begin{aligned} \min _{x \in \mathbb {R}^n}{\left\{ \frac{1}{2}x^TKx + C\sum _{i=1}^n \max \{1-(Kx)_iY_i,0\} \right\} }. \end{aligned}$$

Here, $C>0$ denotes the regularization parameter controlling the trade-off between the loss function and the regularization term. Hence, in order to determine the decision function we solved the convex optimization problem (33), which can be written as

$$\begin{aligned} \min _{x \in \mathbb {R}^n} \left\{ f(x)+g(Kx)\right\} \end{aligned}$$

or, equivalently,

$$\begin{aligned} \min _{x \in \mathbb {R}^n, z \in \mathbb {R}^n}&\left\{ f(x)+g(z)\right\} , \text { s.t. } Kx-z=0 \end{aligned}$$

where $f:\mathbb {R}^n\rightarrow \mathbb {R}, f(x)=\frac{1}{2}x^TKx$, and $g:\mathbb {R}^n \rightarrow \mathbb {R}$ is defined by $g(z)=C\sum _{i=1}^{n}\max \{1-z_iY_i,0\}$.

Since the Gram matrix *K* is positively definite, the function *f* is $\lambda _{\min }(K)$-strongly convex, where $\lambda _{\min }(K)$ denotes the minimal eigenvalue of *K*, and differentiable, and it holds $\nabla f(x)= Kx$ for all $x \in \mathbb {R}^n$. For an element of the form $p = (p_1,\ldots ,p_n) \in \mathbb {R}^n$, it holds

$$\begin{aligned} g^*(p)={\left\{ \begin{array}{ll} \sum _{i=1}^{n}p_iY_i,&{}\quad \text {if } p_iY_i \in [-C,0],\quad i=1, \ldots , n,\\ +\infty , &{}\quad \text {otherwise.} \end{array}\right. } \end{aligned}$$

Consequently, for every $\mu >0$ and $p = (p_1,\ldots ,p_n) \in \mathbb {R}^n$, it holds

$$\begin{aligned} \text {Prox}_{\mu g^*}(x)=\left( \mathcal {P}_{Y_1[-C,0]}(p_1-\sigma Y_1),\ldots ,\mathcal {P}_{Y_n[-C,0]}(p_n-\sigma Y_n)\right) , \end{aligned}$$

where $\mathcal {P}_{Y_i[-C,0]}$ denotes the projection operator on the set $Y_i[-C,0], i=1,\ldots ,n$.

We implemented Proximal AMA for $M_2^k=0$ for all $k \ge 0$ and different choices for the sequence $(M_1^k)_{k \ge 0}$. This resulted in an iterative scheme which reads for all $k \ge 0$:

34 $$\begin{aligned} x^{k+1}&={{\,\mathrm{argmin}\,}}_{x \in \mathbb {R}^n}\left\{ f(x)-\langle p^k,K x \rangle + \frac{1}{2}\Vert x-x^{k}\Vert ^2_{M_1^{k}}\right\} =(K+M_1^k)^{-1}(Kp^k+M_1^kx^{k}) \end{aligned}$$

35 $$\begin{aligned} z^{k+1}&=\text {Prox}_{\frac{1}{c_k}g}\left( Kx^{k+1}-\frac{1}{c^k}p^k\right) =\left( Kx^{k+1}-\frac{1}{c^k}p^k\right) -\frac{1}{c_k}\text {Prox}_{c_k g^*}\left( c_kKx^{k+1}-p^k\right) \nonumber \\ p^{k+1}&=p^k+c_k(-K x^{k+1}+ z^{k+1}). \end{aligned}$$

We would like to emphasize that the AMA method updates the sequence $(z^{k+1})_{k \ge 0}$ also via (35), while the sequence $(x^{k+1})_{k \ge 0}$, as $M_1^k=0$, is updated via $x^{k+1} = p^k$ for all $k \ge 0$. However, it turned out that the Proximal AMA where $M_1^k=\tau _k K,$ for $\tau _k>0$ and all $k \ge 0,$ performs better than the version with $M_1^k=0$ for all $k \ge 0$, which actually corresponds to the AMA method. In this case, (34) becomes $x^{k+1}=\frac{1}{1+\tau _k} (p^k+\tau _kx^{k})$ for all $k \ge 0$.

We used for both algorithms a constant sequence of stepsizes given by $c_k=2 \cdot \frac{\lambda _{\min }(K)}{\Vert K\Vert ^2}-10^{-8}$ for all $k \ge 0$. Tables 1 and 2 show for $C=1$ and different values of the kernel parameter $\sigma$ that Proximal AMA outperforms AMA in what concerns the time and the number of iterates needed to achieve a certain value for a given fixed misclassification rate (which proved to be the best one among several obtained by varying *C* and $\sigma$) and for the RMSE (root-mean-square deviation) for the sequence of primal iterates.

**Table 1:** Performance evaluation of Proximal AMA (with $\tau _k=10$ for all $k \ge 0$) and AMA for the classification problem with $C=1$ and $\sigma =0.2$

Algorithm	Misclassification rate at 0.7027%	RMSE $\le 10^{-3}$
Proximal AMA	8.18 s (145)	23.44 s (416)
AMA	8.65 s (153)	26.64 s (474)

The entries refer to the CPU times in seconds and the number of iterations

**Table 2:** Performance evaluation of Proximal AMA (with $\tau _k=102$ for all $k \ge 0$) and AMA for the classification problem with $C=1$ and $\sigma =0.25$

Algorithm	Misclassification rate at 0.7027%	RMSE $\le 10^{-3}$
Proximal AMA	141.78 s (2448)	629.52 s (10,940)
AMA	147.99 s (2574)	652.61 s (11,368)

The entries refer to the CPU times in seconds and the number of iterations

## Perspectives and Open Problems

In future, it might be interesting to:

1. carry out investigations related to the convergence rates for both the iterates and objective function values of Proximal AMA; as emphasized in [10] for the Proximal ADMM algorithm, the use of variable metrics can have a determinant role in this context, as they may lead to dynamic stepsizes which are favourable to an improved convergence behaviour of the algorithm (see also [15, 21]);
2. consider a slight modification of Algorithm 3.1, by replacing (11) with
  $$\begin{aligned} p^{k+1} = p^k+\theta c_k(b-Ax^{k+1}-Bz^{k+1}), \end{aligned}$$
  where $0<\theta < \frac{\sqrt{5}+1}{2}$ and to investigate the convergence properties of the resulting scheme; it has been noticed in [22] that the numerical performances of the classical ADMM algorithm for convex optimization problems in the presence of a relaxation parameter with $1<\theta <\frac{\sqrt{5}+1}{2}$ outperform the ones obtained when $\theta =1$;
3. embed the investigations made in this paper in the more general framework of monotone inclusion problems, as it was recently done in [10] starting from the Proximal ADMM algorithm.

## Conclusions

The Proximal AMA method has the advantage over the classical AMA method that, as long as the sequence of variable metrics is chosen appropriately, it performs proximal steps when calculating new iterates. In this way, it avoids the use in every iteration of minimization subroutines. In addition, it handles properly smooth and convex functions which might appear in the objective. The sequences of generated iterates converge to a primal–dual solution in the same setting as for the classical AMA method. The fact that instead of solving of minimization subproblems one has only to make proximal steps, may lead to better numerical performances, as we show in the experiments on image processing and support vector machines classification.
